# Structure and Properties of Cellulose/Mycelium Biocomposites

**DOI:** 10.3390/polym14081519

**Published:** 2022-04-08

**Authors:** Adeliya Sayfutdinova, Irina Samofalova, Artem Barkov, Kirill Cherednichenko, Denis Rimashevskiy, Vladimir Vinokurov

**Affiliations:** 1 Department of Physical and Colloidal Chemistry, Gubkin University, 119991 Moscow, Russia; irinasam2014@yandex.ru (I.S.); mycolab@mail.ru (A.B.); 2 Department of Traumatology and Orthopedics, RUDN University, 117198 Moscow, Russia; drimashe@yandex.ru

**Keywords:** microfibrillar cellulose, nanofibrillar cellulose, mycelium-based composites, *Trametes hirsuta*, biobased material, fungi

## Abstract

The current environmental problems require the use of low-energy, environmentally friendly methods and nature-like technologies for the creation of materials. In this work, we aim to study the possibility of the direct biotransformation of fibrillar cellulose by fungi through obtaining a cellulose/mycelium-based biocomposite. The cellulose micro- and nanofibrils were used as the main carbon sources in the solid-phase cultivation of basidiomycete *Trametes hirsuta*. The cellulose fibrils in this process act as a template for growing mycelium with the formation of well-developed net structure. The biotransformation dynamics of cellulose fibrils were studied with the help of scanning electron microscopy. The appearance of nitrogen in the structure of formed fibers was revealed by elemental analysis and FTIR-spectroscopy. The fibers diameters were estimated based on micrograph analysis and the laser diffraction method. It was shown that the diameter of cellulose fibrils can be tuned by fungi through obtaining cellulose-based mycelium fibers with a narrower diameter-size distribution as compared to the pristine cellulose fibrils. The morphology of the resulting mycelium differed when the micro or nanofibrils were used as a substrate.

## 1. Introduction

The demand to replace synthetic hydrocarbon-based plastics with natural polymeric materials is one of the most important challenges of today’s global economy. Cellulose, as the most abundant biopolymer in nature, is still considered as the main raw material for biocompatible and biodegradable materials [1,2,3]. Its high degree of processing makes it possible to obtain micro- and nanofibrils by mechanical [4], chemical [5,6], and enzymatic treatment [7]. For example, the production of nanofibrillar cellulose (CNF) requires the use of sulfuric acid (heating in 60–65% sulfuric acid) or oxidative treatment (in the presence of oxidation catalysts such as TEMPO) [8,9] to form charged functional groups on the cellulose macromolecule surface [10] in order to reduce the excess costs of their further deep mechanical degradation. The enzymatic treatment of cellulose using cellulases can also be effectively used to pretreat cellulose in nanocellulose production [4,11,12] but is restricted by the high cost of enzymes. Thus, the high-energy cost of deep processing of cellulose is the main obstacle towards cheap and widespread cellulose-based nanomaterials. Direct biotransformation of cellulose fibrils of different sizes into corresponding fungal mycelial fibrils can be considered as an alternative way to the above-mentioned physical and chemical cellulose modification.

The mycelium of fungi has a network structure and consists of tubular filaments called hyphae. Hyphae resemble cellulose fibrils in their morphology: the hyphae diameter of higher fungi is about 1–30 µm depending on the fungal strain and cultivation condition [13] and consists mainly of a mechanically strong chitin-β-glucan complex (CGC). Thus, the production of biopolymer microfibers can be realized without additional chemical modification, and the further reduction of the fibril diameter to the nanoscale will require much less energy consumption.

Recently, the fungal mycelium has attracted increasing interest from a fundamental and applied point of view owing to its low-energy consumption while growing, lack of by-products, and broad potential application (paper and textile industries, biomedicine, etc.) [14,15,16]. The biomaterials based on fungal mycelium are considered as a new alternative to synthetic materials and can be used as individual products [17]. For instance, the investigation of the direct interaction of mycelium with primary human dermal fibroblasts revealed its excellent biocompatibility [18]. Growing the mycelium secretes enzymes to both bind and degrade the substrate. Usually, agricultural and agrochemical industry wastes are common substrates for cultivation [19,20], which limits the use of the resulting fibers and composites in medicine, tissue engineering, and food industry since even a small number of impurities (e.g., lignin, heavy metals salts) are not allowed. Therefore, the direct biotransformation of pure fibrillar cellulose used as the main carbon source into the mycelium of higher fungi is a promising low-energy method to obtain fibers of smaller size with higher specific surface. Thus, it is of particular interest to investigate the influence of morphological features of the substrate (with micro- and nano-sized fibrils) on the characteristics of obtained mycelium-based biocomposite and its growth rate.

To the best of our knowledge, there is a lack of data in the literature on the transformation of pure fibrillar cellulose as the main carbon source during mycelium growth. However, there are studies on mycelium/cellulose composites obtained by their physical mixing. Filipova et al. combined the extracted mycelium *Ganoderma applanatum* fibers with cellulose kraft-fiber or/and hemp fiber. The addition of mycelium fibers resulted in increased air permeability [21]. In other study [22], two methods of composite synthesis were compared. In one case, unmodified softwood was mixed with pure white-rot basidiomycete mycelium and a different quantity of nanofibrillar cellulose. In another case, softwood particles modified with pure mycelium were mixed with nanofibrillar cellulose. The composites obtained according to the second method demonstrated the better physical and mechanical properties, which are associated with the CNF-film formation on the surface of modified wood. Attias et al. also used the cellulose nanofibers to produce composites by adding them on the mycelium growth stage. Liquid-phase cultivation of *T. ochracea* mycelium in the presence of carboxymethylated CNF at various concentrations (0.2 to 2 wt%) resulted in more fibrillar, regular, stiffer, and flatter hyphae upon drying [23]. Thus, the architecture of the resulting mycelial fibers can be controlled by using cellulose fibrils as a substrate for fungal growth.

The aim of this work is to study the biotransformation of fibrillar cellulose into fungi hyphae. Here, we present an eco-friendly method of cellulose fibrils modification using fungi mycelium, resulting in the cellulose-based mycelium biocomposite. The biotransformation dynamics of pure micro- and nanofibrils of cellulose during the cultivation of basidiomycete *Trametes hirsuta* using pure cellulose as the main carbon source was studied for the first time. We showed that the diameter of cellulose fibrils can be tuned by fungi through obtaining cellulose-based mycelium fibers with a narrower diameter-size distribution as compared to the pristine cellulose fibrils; the architecture of the resulting mycelium depends on the choice of micro or nanofibrils as a substrate. The presented ecological, low-energy method may be of interest for obtaining micro- and nanofibrillar materials for various purposes, including for biomedical applications.

## 2. Materials and Methods

### 2.1. Materials

Basidiomycete *Trametes hirsuta* MT-17.24 [24] was supplied from the collection of basidiomycetes from the Laboratory of Mycotechnology for the Oil and Gas Industry, Gubkin Russian State University of Oil and Gas. Cellulose pulp (Arkhangelsk Pulp and Paper Mill, Arkhangelsk, Russia), CNF (Manufacturing Co., Ltd., Shinzaike, Japan), NH_4_NO_3_, NaNO_3_ (Merck KGaA, Darmstadt, Germany), and (NH_4_)_2_SO_4_ (BASF, Ludwigshafen, Germany). Yeast Extract Powder, Casein Hydrolysate (Acid) (Oxoid, Basingstoke, UK), and Peptone (Research Center of Pharmacotherapy, Saint-Petersburg, Russia).

### 2.2. Methods

#### 2.2.1. Microfibrillar Cellulose (CMF) Production

CMF production was carried out according to the method described by (Qua et al., 2011) with minor modifications. Instead of the flax fibers, we used the cellulose pulp as the raw material. Before modification, the cellulose pulp was ground in a Bork J500 coffee grinder (Bork, Moscow, Russia) at 600 W. To remove lignin residue and discoloration, the ground cellulose pulp was boiled in hydrogen peroxide, then hydrolyzed in 65% sulfuric acid at 60 °C for 50 min, with 20:1 acid to pulp ratio. The mixture was repeatedly centrifuged to remove acid excess. Precipitate was dispersed in distilled water and then the obtained suspension was sonicated for 30 min at 20 kHz with the help of the Branson Digital Sonifier S-450D (Branson Ultrasonics Corporation, Brookfield, CT, USA). The supernatant was replaced by water to stop the hydrolysis. Centrifugation was repeated until the supernatant became turbid. The suspension was filtered, concentrated, and dried.

#### 2.2.2. Biotransformation of Microfibrillar Cellulose (CMF) and Nanofibrillar Cellulose (CNF)

The growth of *Trametes hirsuta* mycelium on fibrillar cellulose was performed by solid-phase cultivation. Two grams of carbon source (CMF or CNF) was ground before using and placed homogeneously on the Petri dish. A varied quantity of nitrogen source was dissolved in deionized water and added to Petri dish by dropwise. Then, the Petri dish with wetted cellulose fibrils was sterilized for 45 min at 1.5 atm and 121 °C, 3 mm × 3 mm agar fragments with basidiomycete *Trametes hirsuta* mycelium of the collection fungi culture were placed to the center of the Petri dish with the sample, and the sample was incubated in a thermostat at 28 °C. The mycelium growth proceeded for 2 weeks. We stopped the fungal growth at a certain time interval by drying mycelium before investigation. The nitrogen sources used for the study are presented in Table S1 (in Supplementary Materials).

#### 2.2.3. Elemental Analysis

Elemental analysis of C, H, and N was carried out using an Elemental Analyzer (Carlo Erba Strumentazione, Milan, Italy).

#### 2.2.4. Fourier-Transform Infrared Spectroscopy (FTIR)

FTIR-spectra recording was performed using a Nicolet iS 10 FTIR Spectrometer (Thermo Scientific, Waltham, MA, USA) at a range of 608–4000 cm^−1^.

#### 2.2.5. Scanning Electron Microscopy (SEM)

The surface morphology of the samples and biotransformation dynamics were studied with the help of the scanning electron microscope JIB-4501 multibeam system (JEOL, Tokyo, Japan) at voltage acceleration of 10–15 kV.

#### 2.2.6. Evaluation of the Biotransformation Degree

The degree of biotransformation was estimated visually by the disappearance of fibrils larger than 4 microns in diameter. The fibril diameters were measured based on SEM micrographs using freely available ImageJ software (Wayne Rasband, Bethesda, MD, USA).

#### 2.2.7. Laser Diffraction

The particle size distribution of fibril suspensions was measured using a Mastersizer 3000 (Malvern, Worcestershire, UK). Each sample was measured in triplicate and averaged. Before measurement, the CMF, CNF, and the cellulose modified by fungi (CMF_modified_) were ground, and then suspensions were produced in deionized water. CMF and CNF suspensions were sonicated for 30 s at 20 kHz using Branson Digital Sonifier S-450D (Branson Ultrasonics Corporation, Brookfield, CT, USA) before measurement, and CMF_modified_ suspension was measured without additional pre-treatment.

#### 2.2.8. Thermogravimetry Analysis (TGA)

Thermal properties of the samples were studied with a simultaneous thermal analyzer STA 449 F3 Jupiter (NETZSCH, Selb, Germany) in an air atmosphere with a heating rate of 20 °C per min from 0 to 500 °C.

## 3. Results and Discussion

### 3.1. Biotransformation of Fibrillar Cellulose Using Trametes hirsuta

The cellulose enzymatic biotransformation by basidiomycetes during their growth is described elsewhere [25,26,27,28]. As a result of such modification, the fungal hyphae are formed. Peptone, corn starch, and soybean meal are the best nitrogen sources considerably facilitating intensive fungal growth on microfibrillar cellulose. The growth intensity of basidiomycete *Trametes hirsuta* on the substrate was evaluated by visual observations.

We observed a particular correlation between fungi growth and the nature of the nitrogen source and its amount: the basidiomycete was successfully cultivated in the presence of peptone, corn starch, and soybean meal, while in the presence of NaNO_3_, NH_4_NO_3_, (NH_4_)_2_SO_4_, yeast extract, casein hydrolysate, and a mixture of (NH_4_)_2_SO_4_ with peptone, the fungal growth was not observed. A change of cellulose: nitrogen source ratio from 4:1 to 10:1 led to more intense and uniform growth of mycelium, with the best growth occurring on peptone. The obtained mycelium during the solid-phase cultivation is a cotton-like substance of creamy color with a typical mushroom odor. The macrophotograph of biotransformed cellulose is presented in Figure S1 (in Supplementary Materials).

### 3.2. Biotransformation Dynamics of Fibrillar Cellulose

**Figure 1:**
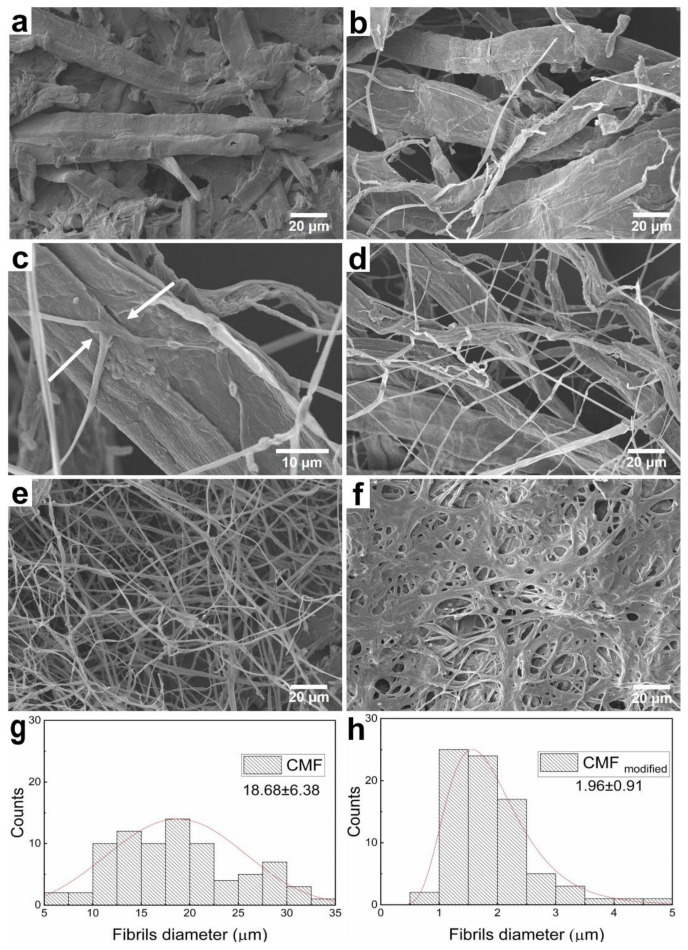
The biotransformation of microfibrillar cellulose: pristine CMF (**a**); mycelium cultivation: 3 days (**b**); hyphae growth from the microfibril structure (arrows indicate the fungi attachment) (**c**); 1 week (**d**); 2 weeks (**e**); 3 weeks (**f**); diameter size distribution: pristine cellulose (**g**); and CMF_modified_ (**h**).

The dynamics of mycelium growth on micro- and nanocellulose fibrils and the morphology of the resulting mycelium were investigated by scanning electron microscopy. The main advantage of using fibrillar cellulose as a substrate for basidiomycete cultivation is the direct control of the resulting mycelium architecture. In this case, the fibrils act as a template for mycelium production. The fungal growth occurs strictly on the fiber surface, which ensures a uniform and fast growth. The biotransformation dynamics of microfibrillar cellulose is presented in SEM micrographs in Figure 1.

After three days of cultivation, we observed successful attachment and colonization of basidiomycete on the cellulose microfibrils (Figure 1b). It can be seen in Figure 1c that the three hyphae are formed simultaneously from the cellulose fiber at an angle of about 90 degrees. After one week of solid-phase cultivation, a partial replacement of the cellulose fibrils by hyphae is observed, which indicates current biotransformation (Figure 1d). The process of anastomosis associated with hyphae merging when they meet allows for the production of branched network mycelium structure during basidiomycete growth [29]. Two weeks later, the formation of a well-developed mycelium net is observed, with initial cellulose being completely transformed into mycelium (Figure 1e). Three weeks of cultivation resulted in the formation of a mycelium sponge-like structure also known as fungal skin (Figure 1f) [19]. In terms of morphology, 1 and 2 weeks of mycelium cultivation are optimal for the production of biocomposites. The biotransformation is accompanied by a reduction in the fibrils diameter from about 20 to 2 μm, indicating the replacement of cellulose fibers by mycelium hyphae, and characterized by a narrower diameter size distribution as compared to initial cellulose fibers. Diameter size distribution before and after 2 weeks of biotransformation is shown in Figure 1g,h.

**Figure 2:**
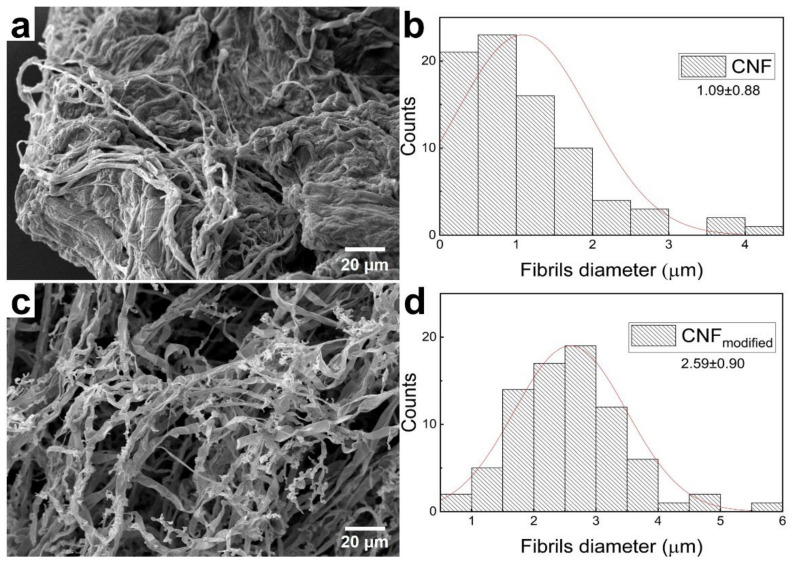
Biotransformation of CNF: pristine CNF (**a**), fibril diameter size distribution (**b**), 5 days of mycelium cultivation (**c**), and fibril diameter size distribution (**d**).

It should be noted that the biotransformation of cellulose fibrils can occur both with a decrease and an increase in the diameter of the resulting fibers, depending on the diameter of the initial fibrils. We also performed the cultivation of basidiomycete *Trametes hirsuta* on CNF. The morphology of pristine CNF and resulting mycelium is shown in SEM micrographs in Figure 2.

The complete replacement of nanofibrils by the fungi hyphae occurred in 5 days, with the diameter being increased from 1 μm to 3 μm. The morphology and growth rate of mycelium on CMF and CNF depend on the substrate volume to be transformed. In the case of nanofibrils, the biotransformation proceeds faster due to the small diameter of the used fibers; herewith, the fungi have to increase their mass to form hyphae. The biotransformation of microfibrils requires more time. When CNF is used as a substrate for cultivation, the distance between the formed hyphae is shorter since the increase of a fiber diameter by an order of magnitude leads to a free volume decrease. Accordingly, when CMF is used, the distance between hyphae increases. Thus, it is possible to tune the architecture and the density of the resulting mycelium in the nanoscale by using different kinds of cellulose.

### 3.3. Laser Diffraction

**Figure 3:**
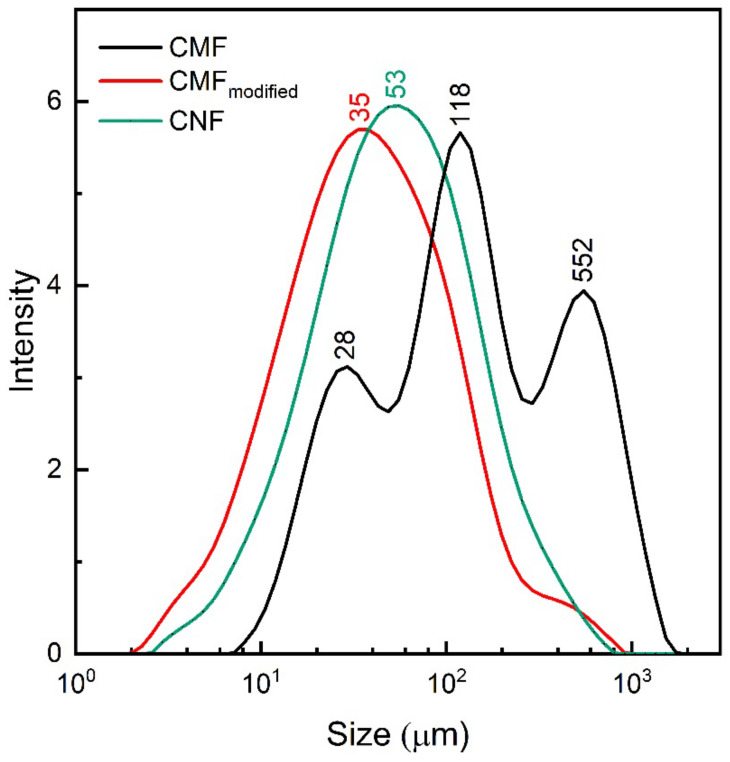
Particle size distribution of the samples: CMF (black), CMF_modified_ (red), and CNF (green).

Although SEM analysis gives useful information about the diameter of the formed hyphae during cellulose biotransformation, the correctness of the measured fibers’ size distribution is limited by a small fraction of analyzed diameters in regard to the whole sample. To obtain more general information about fibril size distribution, the laser diffraction method was employed. It is worth noting that this method strongly depends on particle shape, volume, and concentration. Hence, since the fibrils are not spherical in shape, the obtained size values can considerably differ from those measured in SEM-micrographs. Nevertheless, the laser diffraction method has been already successfully applied to compare the different size distribution of cellulose nanofibers [30]. Here, we compare the particle size distribution of pristine CMF and biotransformed CMF (CMF_modified_) and conclude the effectiveness of biotransformation as a method for producing the fibers of smaller size. As it follows from Figure 3 and Table S2 (in Supplementary Materials), pristine CMF has inhomogeneous particle size distribution: two coarse fractions (118 μm and 552 μm) and small fraction (28 μm). CMF_modified_ shows homogeneous particle size distribution with the small fibril size (35 μm). It should be noted that the cellulose modified by the proposed fungi method is characterized by the narrower fibrils size distribution and the smallest fibrils size (35 μm), as compared to commercially available nanocellulose (53 μm). Thus, biotransformation allows for a decreasing fibrils diameter without any additional chemical or mechanical treatment, which is often non-ecological and requires high energy consumption.

### 3.4. The Composition Characterization

The biotransformation of CMF fibrils during fungi growth results in the formation of fungi hyphae with different chemical compositions. The cell walls of basidiomycetes contain chitin, an alkali- and acid-resistant glucan; proteins; etc. The chitin-containing end product obtained from fungi appears as a chitin–glucan complex (CGC) [31], hence the CMF_modified_ should contain chitin and/or CGC, which was confirmed by elemental analysis and FTIR. The elemental compositions of CMF_modified_, pristine CMF, and commercial crustacean chitin are compared in Table 1.

Biotransformed CMF contains approximately 1% of the nitrogen in the structure, which is associated with non-bonded chitin, chitin-glucan, and protein. For example, nitrogen content in pure CGC extracted from basidiomycete *Schizophyllum commune* is 1.22 [32]. Commercial crustacean chitin contains 5.5% of nitrogen, which is comparable with 7% reported by [33].

**Figure 4:**
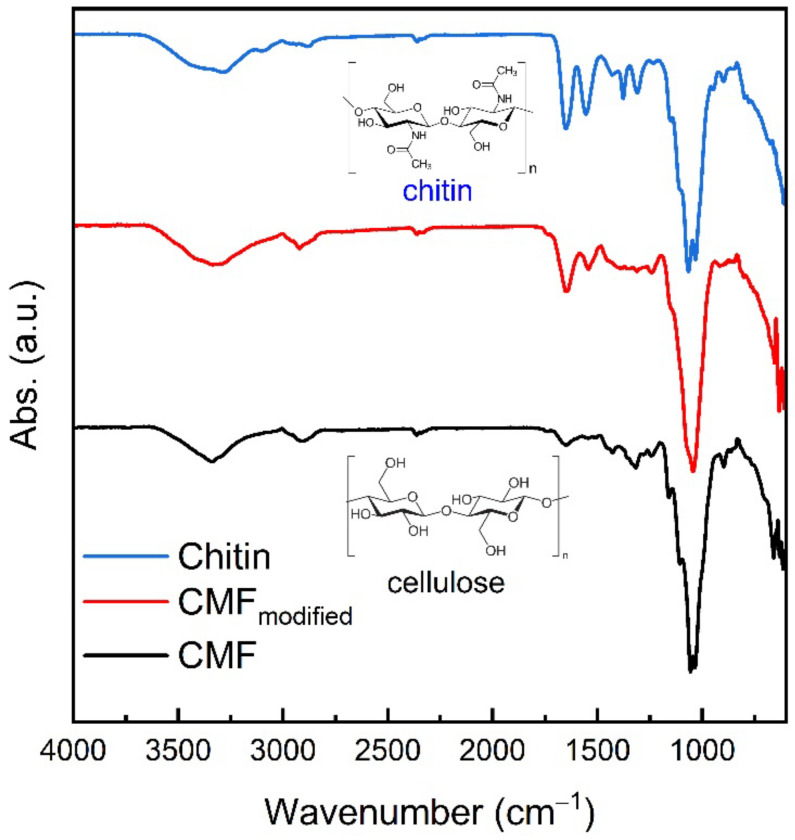
FTIR-spectra of pristine CMF, CMF_modified_, and crustacean chitin.

The results obtained from elemental analysis are in good agreement with FTIR data. The FTIR-spectra of CMF, CMF_modified_, and chitin are presented in Figure 4. The well-known cellulose and chitin characteristic peaks are presented in a number of publications [30,34,35,36]. The broad intense band in the range of 3500 and 3300 cm^−1^ presented in all spectra refers to the hydroxyl group vibration indicating the presence of intra- and intermolecular interactions of the polysaccharide chains. In CMF_modified_ and chitin spectra, this band overlaps with N-H amide stretching vibration in the range of 3270–3100 cm^−1^. CMF_modified_ and chitin FTIR spectra also contain an amide group at the C_2_ atom, which appears as Amide I-III adsorption bands. The peak of 1645 cm^−1^ corresponding to absorbed O-H is observed for cellulose and overlaps with Amide I stretching vibrations of C=O bonds of carbonyl groups in acetamides revealed at 1647 cm^−1^ and 1650 cm^−1^ for CMF_modified_ and chitin correspondingly. Amide II bands assigned to the vibrations of N–H bond in amides were observed in CMF_modified_ and chitin spectra at 1543 cm^−1^ and 1556 cm^−1^ correspondingly. The adsorption bands of CMF_modified_ and chitin at 1313 cm^−1^ and 1311 cm^−1^ resulted from C–N deformation, while 1317 cm^−1^ band of CMF was attributed to in-plane CH_2_ deformation. The intensive peak at the range of 1150–920 cm^−1^ confirms the presence of pyranose ring in polysaccharides. The main characteristic peaks revealed in spectra are presented in Table S3 (in Supplementary Materials).

### 3.5. Thermal Properties

**Figure 5:**
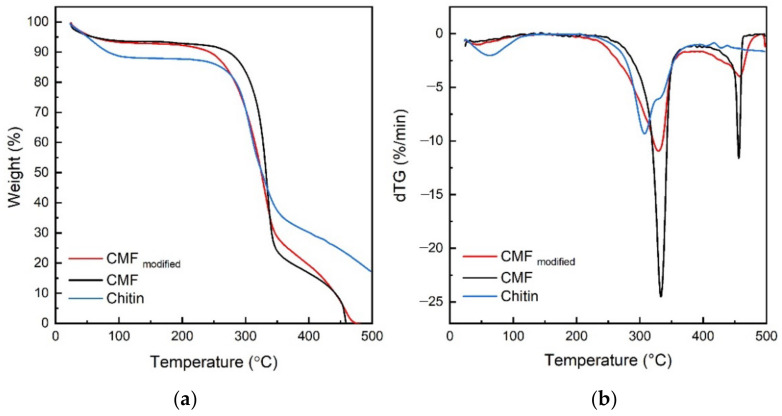
TGA (**a**) and dTG (**b**) curves of CMF_modified_, CMF, and chitin.

The thermal properties of CMF_modified_, CMF, and chitin were investigated (Figure 5). The mass loss of chitin up to 10% in the range of 30 to 130 °C is associated with water evaporation [37]. The main mass loss of all samples (∆~60%) occurred within 250–350 °C range and is attributed to the destruction of saccharide structure, as well as to the decomposition of acetylated units of chitin in CMF_modified_ and chitin [38]. Chitin is less resistant than cellulose to thermal stress, so the decomposition temperatures T_deg_ decrease in the range of CMF > CMF_modified_ > chitin and are 333 > 330 > 308 °C, correspondingly. For instance, the maximum decomposition temperature of mycelium films produced in [39] is reached at 300 °C. At the temperatures higher than 350 °C, a monotonic mass loss is observed for all samples.

## 4. Conclusions

We performed the biotransformation of cellulose during fungal growth in which cellulose was the main carbon source, thus resulting in the formation of cellulose-based mycelium fibers. The optimal ratio of carbon:nitrogen sources facilitating more intense and uniform fungal growth was 10 to 1. The complete transformation of cellulose microfibrils into basidiomycetes hyphae occurs during 2-weeks of solid-phase cultivation, whereas complete biotransformation of nanofibrils takes 5 days. The micro and nanofibrils of cellulose act as a template for forming mycelium. A branched network of fungal mycelium appears in place of the cellulose microfibrils, and the size of the resulting hyphae differs by an order of magnitude from the size of the initial microfibrils, which is also confirmed by laser diffraction. A suspension of cellulose-based mycelium fibers in water is characterized by the smallest fibril size and narrower fibril size distribution compared to the suspension of initial cellulose and is comparable to the fibril size of commercial nanocellulose suspension. Thus, biotransformation allows for a decreasing fibrils diameter without any additional chemical or mechanical treatment. FTIR-spectrum of modified cellulose differs from that of the initial cellulose by the appearance of characteristic bands at 1647 cm^−1^, 1543 cm^−1,^ and 1313 cm^−1^ owing to the acetamide group in the chitin/chitin glucan contained in the cell wall of the formed fungi fibers. The FTIR data are in good agreement with elemental analysis data.

The presented method of obtaining fibers on the basis of cellulose and mycelium is environmentally friendly and low-energy-consuming and can be used for the creation of micro- and nanofibrillar materials for various purposes. Since cellulose and mycelium are both biocompatible and non-toxic materials, there are strong grounds to believe that they can be applied in biomedical applications. Nevertheless, this requires a separate comprehensive investigation.

## Figures and Tables

**Table 1:** Elemental analysis of CMF_modified_, pristine CMF, and chitin.

Biopolymer	C, %	H, %	N, %
CMF	44.03 ± 0.06	6.42 ± 0.07	0
CMF_modified_	43.78 ± 0.12	6.29 ± 0.01	1 ± 0.10
Chitin (Crustaceans)	37.3 ± 0.08	6.09 ± 0.07	5.5 ± 0.03

## Data Availability

The data presented in this study are available on request from the corresponding author.

## Supplementary Materials

The following supporting information can be downloaded at: https://www.mdpi.com/article/10.3390/polym14081519/s1, Table S1: Nitrogen sources are chosen for biotransformation; Table S2: The fibrils size distribution and percentile values of the different fibrils in suspensions; Table S3: FTIR spectral values of main groups in CMF, CMF_modified_, and chitin; Figure S1: The macrophotograph of biotransformed cellulose into fungi mycelium.

## References

[1] Fatima A., Yasir S., Khan M.S., Manan S., Ullah M.W., Ul-Islam M. (2021). Plant Extract-Loaded Bacterial Cellulose Composite Membrane for Potential Biomedical Applications. J. Bioresour. Bioprod..

[2] Acharya S., Liyanage S., Abidi N., Parajuli P., Rumi S.S., Shamshina J.L. (2021). Utilization of Cellulose to Its Full Potential: A Review on Cellulose Dissolution, Regeneration, and Applications. Polymers.

[3] Ahmad F., Mushtaq B., Butt F.A., Zafar M.S., Ahmad S., Afzal A., Nawab Y., Rasheed A., Ulker Z. (2021). Synthesis and Characterization of Nonwoven Cotton-Reinforced Cellulose Hydrogel for Wound Dressings. Polymers.

[4] Souza A.G., Santos D.F., Ferreira R.R., Pinto V.Z., Rosa D.S. (2020). Innovative Process for Obtaining Modified Nanocellulose from Soybean Straw. Int. J. Biol. Macromol..

[5] Abdul Khalil H.P.S., Davoudpour Y., Islam M.N., Mustapha A., Sudesh K., Dungani R., Jawaid M. (2014). Production and Modification of Nanofibrillated Cellulose Using Various Mechanical Processes: A Review. Carbohydr. Polym..

[6] Dhali K., Ghasemlou M., Daver F., Cass P., Adhikari B. (2021). A Review of Nanocellulose as a New Material towards Environmental Sustainability. Sci. Total Environ..

[7] Bauli C.R., Rocha D.B., de Oliveira S.A., Rosa D.S. (2019). Cellulose Nanostructures from Wood Waste with Low Input Consumption. J. Clean. Prod..

[8] Weishaupt R., Siqueira G., Schubert M., Tingaut P., Maniura-Weber K., Zimmermann T., Thöny-Meyer L., Faccio G., Ihssen J. (2015). TEMPO-Oxidized Nanofibrillated Cellulose as a High Density Carrier for Bioactive Molecules. Biomacromolecules.

[9] Puangsin B., Soeta H., Saito T., Isogai A. (2017). Characterization of Cellulose Nanofibrils Prepared by Direct TEMPO-Mediated Oxidation of Hemp Bast. Cellulose.

[10] Peyre J., Pääkkönen T., Reza M., Kontturi E. (2015). Simultaneous Preparation of Cellulose Nanocrystals and Micron-Sized Porous Colloidal Particles of Cellulose by TEMPO-Mediated Oxidation. Green Chem..

[11] Chen X.Q., Deng X.Y., Shen W.H., Jia M.Y. (2018). Preparation and Characterization of the Spherical Nanosized Cellulose by the Enzymatic Hydrolysis of Pulp Fibers. Carbohydr. Polym..

[12] Michelin M., Gomes D.G., Romaní A., Polizeli M.d.L.T.M., Teixeira J.A. (2020). Nanocellulose Production: Exploring the Enzymatic Route and Residues of Pulp and Paper Industry. Molecules.

[13] Islam M.R., Tudryn G., Bucinell R., Schadler L., Picu R.C. (2017). Morphology and Mechanics of Fungal Mycelium. Sci. Rep..

[14] Yang L., Park D., Qin Z. (2021). Material Function of Mycelium-Based Bio-Composite: A Review. Front. Mater..

[15] Jones M., Mautner A., Luenco S., Bismarck A., John S. (2020). Engineered Mycelium Composite Construction Materials from Fungal Biorefineries: A Critical Review. Mater. Des..

[16] Jones M., Huynh T., Dekiwadia C., Daver F., John S. (2017). Mycelium Composites: A Review of Engineering Characteristics and Growth Kinetics. J. Bionanosci..

[17] Sydor M., Bonenberg A., Doczekalska B., Cofta G. (2021). Mycelium-Based Composites in Art, Architecture, and Interior Design: A Review. Polymers.

[18] Antinori M.E., Contardi M., Suarato G., Armirotti A., Bertorelli R., Mancini G., Debellis D., Athanassiou A. (2021). Advanced Mycelium Materials as Potential Self-Growing Biomedical Scaffolds. Sci. Rep..

[19] Zimele Z., Irbe I., Grinins J., Bikovens O., Verovkins A., Bajare D. (2020). Novel Mycelium-Based Biocomposites (MBB) as Building Materials. J. Renew. Mater..

[20] Antinori M.E., Ceseracciu L., Mancini G., Heredia-Guerrero J.A., Athanassiou A. (2020). Fine-Tuning of Physicochemical Properties and Growth Dynamics of Mycelium-Based Materials. ACS Appl. Bio Mater..

[21] Filipova I., Irbe I., Spade M., Skute M., Dāboliņa I., Baltiņa I., Vecbiskena L. (2021). Mechanical and Air Permeability Performance of Novel Biobased Materials from Fungal Hyphae and Cellulose Fibers. Materials.

[22] Sun W., Tajvidi M., Hunt C.G., McIntyre G., Gardner D.J. (2019). Fully Bio-Based Hybrid Composites Made of Wood, Fungal Mycelium and Cellulose Nanofibrils. Sci. Rep..

[23] Attias N., Reid M., Mijowska S.C., Dobryden I., Isaksson M., Pokroy B., Grobman Y.J., Abitbol T. (2021). Biofabrication of Nanocellulose–Mycelium Hybrid Materials. Adv. Sustain. Syst..

[24] Kozhevnikova E.Y., Barkov A.V., Spitsyna E.A., Petrova D.A., Novikov A.A., Kotelev M.S., Gushchin P.A., Ivanov E.V., Vinokurov V.A. (2017). Basidiomycete Strain Trametes Hirsuta.

[25] Coughlan M.P., Fogarty W.M., Kelly C.T. (1990). Cellulose Degradation by Fungi. Microbial Enzymes and Biotechnology.

[26] Baldrian P., Valášková V. (2008). Degradation of Cellulose by Basidiomycetous Fungi. FEMS Microbiol. Rev..

[27] Freitas G.A.D., Almeida A.F.D., Conceição R.C.N.D., Luz J.H.S.D., Nunes B.H.D.N., Ribeiro E.A., Ribeiro F.D.S., Neto A.A., Machado Â.F., Deusdará T.T. (2019). Fungi with Cellulolytic Potential: Screening, Inoculum, and Methodology for Isolation. Int. J. Adv. Eng. Res. Sci..

[28] Várnai A., Mäkelä M.R., Djajadi D.T., Rahikainen J., Hatakka A., Viikari L. (2014). Carbohydrate-Binding Modules of Fungal Cellulases. Adv. Appl. Microbiol..

[29] Angelova G.V., Brazkova M.S., Krastanov A.I. (2021). Renewable Mycelium Based Composite—Sustainable Approach for Lignocellulose Waste Recovery and Alternative to Synthetic Materials—A Review. Z. Naturforsch. Sect. C J. Biosci..

[30] Qua E.H., Hornsby P.R., Sharma H.S.S., Lyons G. (2011). Preparation and Characterisation of Cellulose Nanofibres. J. Mater. Sci..

[31] Kim H., Kang S., Li K., Jung D., Park K., Lee J. (2021). Preparation and Characterization of Various Chitin-Glucan Complexes Derived from White Button Mushroom Using a Deep Eutectic Solvent-Based Ecofriendly Method. Int. J. Biol. Macromol..

[32] Smirnou D., Krcmar M., Prochazkova E. (2011). Chitin-Glucan Complex Production by Schizophyllum Commune Submerged Cultivation. Pol. J. Microbiol..

[33] Roca C., Chagas B., Farinha I., Freitas F., Mafra L., Aguiar F., Oliveira R., Reis M.A.M. (2012). Production of Yeast Chitin–Glucan Complex from Biodiesel Industry Byproduct. Process Biochem..

[34] Hong Y., Ying T. (2019). Characterization of a Chitin-Glucan Complex from the Fruiting Body of *Termitomyces albuminosus* (Berk.) Heim. Int. J. Biol. Macromol..

[35] Boureghda Y., Satha H., Bendebane F. (2021). Chitin–Glucan Complex from *Pleurotus ostreatus* Mushroom: Physicochemical Characterization and Comparison of Extraction Methods. Waste Biomass Valorizat..

[36] Ferreira I.C., Araújo D., Voisin P., Alves V.D., Rosatella A.A., Afonso C.A.M., Freitas F., Neves L.A. (2020). Chitin-Glucan Complex—Based Biopolymeric Structures Using Biocompatible Ionic Liquids. Carbohydr. Polym..

[37] Abdel-Rahman R.M., Hrdina R., Abdel-Mohsen A.M., Fouda M.M.G., Soliman A.Y., Mohamed F.K., Mohsin K., Pinto T.D. (2015). Chitin and Chitosan from Brazilian Atlantic Coast: Isolation, Characterization and Antibacterial Activity. Int. J. Biol. Macromol..

[38] Araújo D., Alves V.D., Marques A.C., Fortunato E., Reis M.A.M., Freitas F. (2020). Low Temperature Dissolution of Yeast Chitin-Glucan Complex and Characterization of the Regenerated Polymer. Bioengineering.

[39] César E., Canche-Escamilla G., Montoya L., Ramos A., Duarte-Aranda S., Bandala V.M. (2021). Characterization and Physical Properties of Mycelium Films Obtained from Wild Fungi: Natural Materials for Potential Biotechnological Applications. J. Polym. Environ..

