# Supplementary material for: Structure and Properties of Cellulose/Mycelium Biocomposites
Source: Polymers (Basel). 2022 Apr 8;14(8):1519. doi: 10.3390/polym14081519 (PMC9030294; doi:10.3390/polym14081519)
Supplement: Supplementary file 1 [file polymers-14-01519-s001.zip › polymers-1656206-supplementary.pdf]

polymers

MDPI

Supplementary Materials

Structure and Properties of Cellulose/Mycelium Biocomposites

Adeliya Sayfutdinova [1,*], Irina Samofalova [1], Artem Barkov [1], Kirill Cherednichenko [1,*], Denis Rimashevskiy [2] and Vladimir Vinokurov [1,*]

[1] Department of Physical and Colloidal Chemistry, Gubkin University, 119991 Moscow, Russia; irinasam2014@yandex.ru (I.S.); mycolab@mail.ru (A.B.)
[2] Department of Traumatology and Orthopedics, RUDN University, 117198 Moscow, Russia; drimashe@yandex.ru
* Correspondence: sayfutdinova.a@gubkin.ru (A.S.), cherednichenko.k@gubkin.ru (K.C.), vladimir@vinokurov.me (V.V.)

Table S1. Nitrogen sources are chosen for biotransformation.

Nitrogen Source	Fungi Growth
$NaNO_3$	-*
NH_4NO_3	-
$(NH_4)_2SO_4$	-
Yeast Extract	-
Peptone	+
Corn Extract	+
Soybean meal	+
Casein Hydrolyzate	-
$(NH_4)_2SO_4$ + peptone	-

* The «plus» indicates the successful growth of fungi in the presence of a nitrogen source, the absence of growth is labelled as «minus».

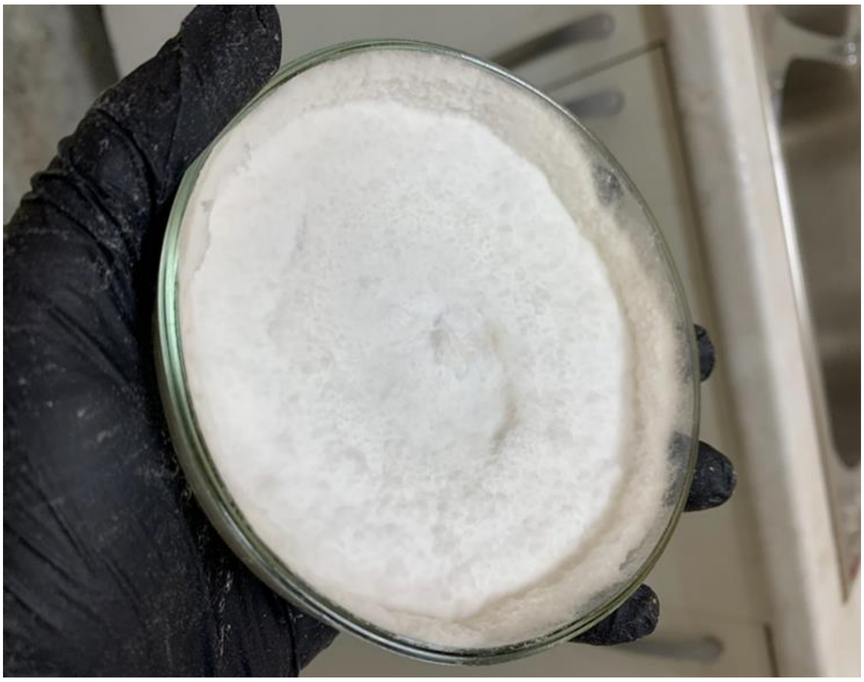

Figure S1. The macrophotograph of biotransformed cellulose into fungi mycelium. (The photo was taken by one of the authors).

Table S2. The fibrils size distribution and percentile values of the different fibrils in suspensions.

Sample	Dx (10) (μm)	Dx (50) (μm)	Dx (90) (μm)	Mode
CMF	24.1	128	672	119.0
CMF $_{modified}$	9.6	37	138	35.3
CNF	14.0	52.5	183	52.5

Table S3. FTIR spectral values of main groups in CMF, CMF $_{modified}$ and chitin.

Vibration Modes	Wavenumber, cm^{-1}			
	**CMF**	**CMF $_{modified}$**	**Chitin**	**Group**
OH stretching	3348	Broad band	Broad band	Glucan, chitin
NH- stretching (asymmetric)<br>O-H···O stretching hydrogen-bonded		3307	3295	Glucan, chitin
NH-stretching (symmetric)			3095	Chitin
C-H stretching of CH_2 and CH_3 groups	2907	2923		Glucan
C-H stretching			2881	Chitin
Adsorbed O-H or C=O<br>Amide I band	1642	1647	1650	Glucan, chitin
Amide II band		1543	1556	Chitin
C-H deformation	1430		1427	Glucan, chitin
C-H bending and symmetric CH_3 deformation		1375	1377	Chitin
In-plane CH_2 deformation<br>Amide III band	1317	1313	1311	Glucan, chitin
Amide IV band		1245	1282	Chitin
O-H in-plane bending and C-O stretch (symmetric)	1203	1205		Glucan, chitin
C-O-C stretching (asymmetric)	1109		1153	Glucan, chitin
C-OH stretching	1057<br>1035	1045	1066<br>1032	Glucan, chitin
Rocking vibration of CH_2	897	875	899	Glucan, chitin
Out of plane O-H···O hydrogen bonding	655	668	656	Glucan, chitin
